# Enhancing Sensitivity in Gas Detection: Porous Structures in Organic Field-Effect Transistor-Based Sensors

**DOI:** 10.3390/s24092862

**Published:** 2024-04-30

**Authors:** Soohwan Lim, Ky Van Nguyen, Wi Hyoung Lee

**Affiliations:** Department of Materials Science and Engineering, School of Chemical Engineering, Konkuk University, Seoul 05029, Republic of Korea

**Keywords:** organic semiconductor, organic field-effect transistors, gas sensor, sensitivity, porous structure, microstructure

## Abstract

Gas detection is crucial for detecting environmentally harmful gases. Organic field-effect transistor (OFET)-based gas sensors have attracted attention due to their promising performance and potential for integration into flexible and wearable devices. This review examines the operating mechanisms of OFET-based gas sensors and explores methods for improving sensitivity, with a focus on porous structures. Researchers have achieved significant enhancements in sensor performance by controlling the thickness and free volume of the organic semiconductor layer. Additionally, innovative fabrication techniques like self-assembly and etching have been used to create porous structures, facilitating the diffusion of target gas molecules, and improving sensor response and recovery. These advancements in porous structure fabrication suggest a promising future for OFET-based gas sensors, offering increased sensitivity and selectivity across various applications.

## 1. Introduction

Organic field-effect transistors (OFETs) have garnered significant attention as a promising candidate for flexible display backplanes [1]. Typically, OFETs consist of an organic semiconductor, gate dielectric, and three electrodes (i.e., source, drain, and gate electrodes), where the gate bias induces polarization in the gate dielectric, leading to an accumulation of charge carriers (e.g., electrons, holes) in the organic semiconductor near the gate dielectric [2]. The potential difference between the source and drain electrodes results in a current flow from the source to the drain electrodes. The magnitude and direction of the gate bias determine the current flow and corresponding switching capability. The carrier type is determined by the energy gap between the Fermi level of the source/drain electrode and the lowest unoccupied molecular orbital (LUMO) or highest occupied molecular orbital (HOMO). P-type OFETs facilitate hole injection from the Fermi level of the source electrode to the HOMO of the organic semiconductor [3,4,5].

**Figure 1:**
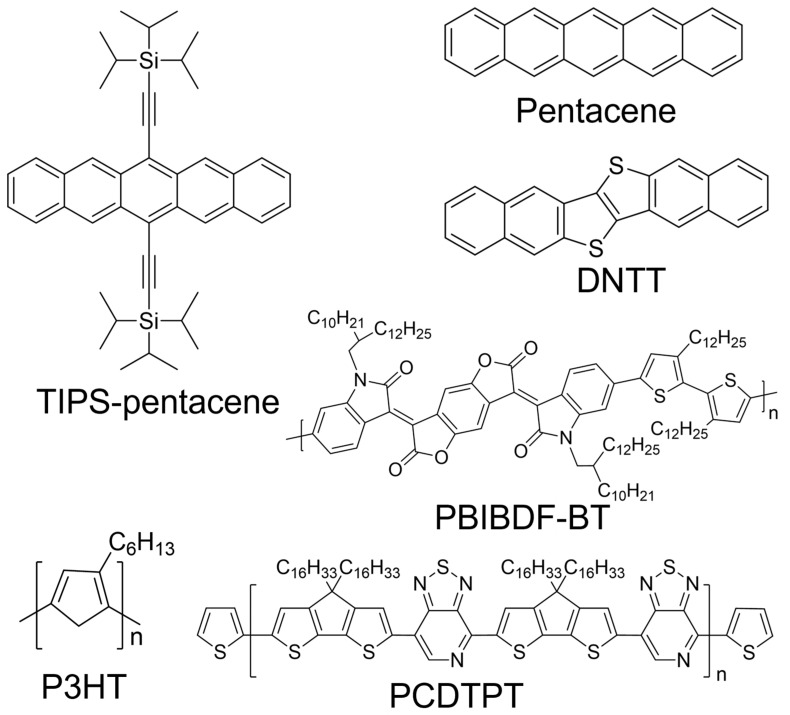
Various organic semiconductors for OFET gas sensors.

Figure 1 illustrates a prototypical organic semiconductor used in p-type OFETs. Pentacene and Dinaphtho [2,3-b:2′,3′-f] thieno [3,2-b] thiophene (DNTT) are small molecule organic semiconductors deposited through thermal evaporation. Acene or hetero-acene structures with extended conjugation are common motifs for enabling extended conjugation while reducing the bandgap in organic semiconductors [6,7]. Since pentacene is not soluble in common organic solvents, the attachment of bulky alkyl groups (e.g., triisopropylsilylethynyl group) can increase its solubility [8]. Thus, synthesized TIPS-pentacene is soluble in organic solvents [9]. Furthermore, the attached bulky group can disturb herringbone stacking, leading to co-facial stacking with minimized π-π stacking distance [10]. However, the microstructural development of TIPS-pentacene is very sensitive to processing conditions, and optimizing solution processing conditions is necessary. On the other hand, polymeric semiconductors such as poly(3-hexylthiophene) (P3HT) can serve as alternative organic semiconductors suitable for low-cost and high-throughput printing processes [11]. Although the carrier mobility of P3HT FETs is typically low, recent advancements in polymeric semiconductors (e.g., bis(2-oxoindolin-3-ylidene)-benzodifuran-dione (PBIBDF-BT), poly [4-(4,4-dihexadecyl-4H-cyclopenta[1,2-b:5,4-b′]dithiophen-2-yl)-alt-[1,2,5]thiadiazolo[3,4-c]pyridine] (PCDTPT) ) can lead to high-performance OFETs [12]. In terms of carrier mobility, recent polymeric semiconductors rival pentacene or TIPS-pentacene, exhibiting carrier mobilities exceeding 1 cm^2^/Vs. Recent review papers have discussed the molecular aspects of synthesized organic semiconductors used in OFETs, providing comparative analyses to determine charge carrier mobility in OFETs [13,14].

In addition to their use in switching display backplanes, OFETs can also serve as sensors for detecting chemical and biological elements. However, organic semiconductors are vulnerable to oxidation in humid conditions, posing challenges for sensing in aqueous environments. Therefore, the gaseous state of the target analyte is preferred, and gas sensors utilizing OFETs could provide an alternative solution. OFET-based gas sensors operate through chemical and physical interactions between gas analytes and semiconductor layers. With numerous organic semiconductors available and various methods for specifically binding gas molecules, OFETs can detect a wide range of target gas molecules. Particularly, environmentally harmful gases such as NO_x_ and NH_3_ can be detected by monitoring the source–drain current with OFETs [4,15,16,17].

In this review paper, we begin by discussing the operating mechanism of OFET-based gas sensors. Then, we review several strategies to enhance gas diffusion for improved sensitivity in OFET gas sensors. The control of thickness and free volume is one way to enhance sensitivity. We will focus on reviewing porous structures that can be fabricated through self-assembly methods or etching. Our paper reviews recent advancements in fabricating various types of porous structures for highly sensitive OFET-based gas sensors.

## 2. OFET-Based Gas Sensors—Operating Mechanism

**Figure 2:**
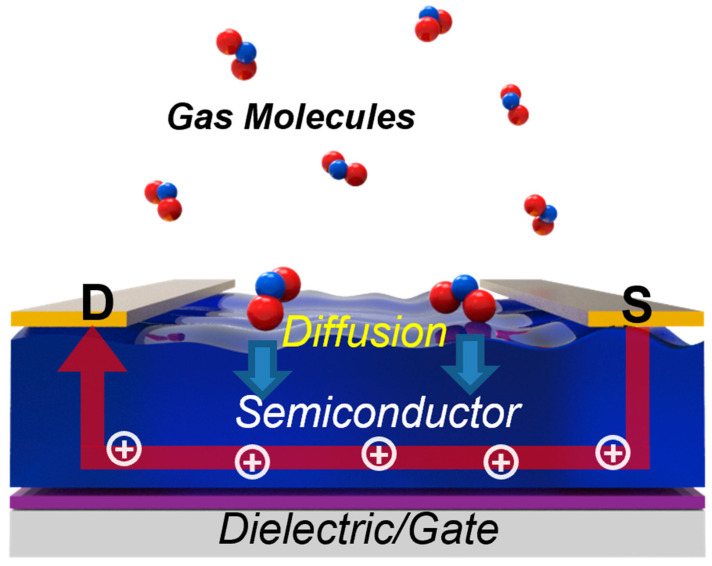
Schematic illustration of the gas-sensing mechanism.

Figure 2 shows the operating mechanism of OFET-based gas sensors. Although the n-type operation of OFET is possible by tuning the LUMO level near the Fermi level of the source–drain electrode, the fabricated OFET typically shows low environmental stability [18,19]. Thus, most OFET gas sensors have been fabricated with p-type OFETs where holes are the major carrier type [20]. Because the carrier density of organic semiconductors is typically low, negative gate bias needs to be applied to induce hole carriers near the gate dielectric [21]. When hole carriers pass through between the source and drain electrodes, the adsorbed gas molecules can affect the current flow. There might be several ways for the effects of the adsorbed gas molecules. One is the scattering effect, which degrades carrier mobility in OFETs [15]. On the other hand, the adsorbed molecule can induce a doping effect, which changes the carrier density in OFETs. Because the electric field in OFETs is the highest at the semiconducting layer near the gate dielectric, the adsorbed gas molecules need to diffuse into the semiconductor–dielectric interface to amplify the scattering of the doping effect [4,22].

When polar gases such as NH_3_ or NO_x_ are present, the diffused gas molecules can induce a dipolar effect, thereby leading to current change. By monitoring current changes in OFETs, it is possible to monitor the concentration of target gas molecules in a given OFET structure [23]. There are several key performance parameters in gas sensing: selectivity, sensitivity, recovery, and stability [24]. Selectivity, also known as cross-sensitivity, refers to the ability to detect target gas molecules within a mixture of various gases. Sensitivity, on the other hand, is the normalized gas response to specific gas molecules. Recovery denotes the sensor’s ability to return to its original signal once the gas molecules are no longer present, while stability pertains to the sensor’s ability to operate effectively over an extended period [25,26].

Numerous well-written review papers exist on achieving selectivity in OFET-based gas sensors [27,28,29]. It is widely acknowledged that OFET-based gas sensors can detect dipolar gas molecules. Notably, charge transfer and accumulation indeed occur between organic semiconductors and gas molecules. Typically, p-type semiconductors are used in OFET-based gas sensors [30,31]. Consequently, NH_3_, with its electron-donating character, can deplete existing hole carriers in p-type semiconductors, leading to a decrease in the current between the source and drain electrodes. Conversely, NO_2_, with its electron-withdrawing character, can accumulate hole carriers, resulting in an increase in current. This dipolar effect is also relevant in explaining the gas-sensing properties of OFETs. The direction and magnitude of the source–drain current can thus be utilized for gas molecule detection [32,33,34,35]. Given that the gas-sensing mechanism is closely tied to changes in the magnitude and mobility of field-effect charge carriers near the semiconductor–dielectric interface, the diffusion of gas molecules within the semiconductor is crucial. Microstructural engineering of the semiconductor layers, including morphology and structure, offers a viable approach to improving sensor performance [36]. Specifically, the enhanced surface roughness of semiconducting layers provides sites for gas adsorption, thereby enhancing sensor performance.

There are several methods to facilitate the diffusion of gas analytes into the active channel of OFETs. The first approach involves creating air dielectric transistors, where the gas analyte is directly in contact with the conductive channel of the semiconductor [37]. This allows for absorption and enhanced gas sensitivity. For instance, in a gas sensor utilizing Copper phthalocyanine (CuPc) as a semiconductor, comparing the sensitivity of two sensors—one with an air dielectric and the other with a PMMA dielectric—reveals a difference of more than 200 times. However, these devices are more challenging to fabricate than typical transistors. Thus, we will not cover this strategy in this review paper. The second approach involves controlling the free volume of organic semiconductors. The third approach is to thin the conductive channel, as many studies have shown that ultrathin films can improve sensing response, recovery, and sensitivity [16,17]. The final approach is to increase the contact between the gas and the conductive channel by fabricating a microporous film. The porous film facilitates gas diffusion, proving to be an effective strategy for enhancing sensor response and recovery. In the following section, we will introduce strategies ranging from controlling thickness and free volume to forming porous structures via self-assembly or etching.

## 3. Control of Thickness and Free Volume for Enhanced Sensitivity

**Figure 3:**
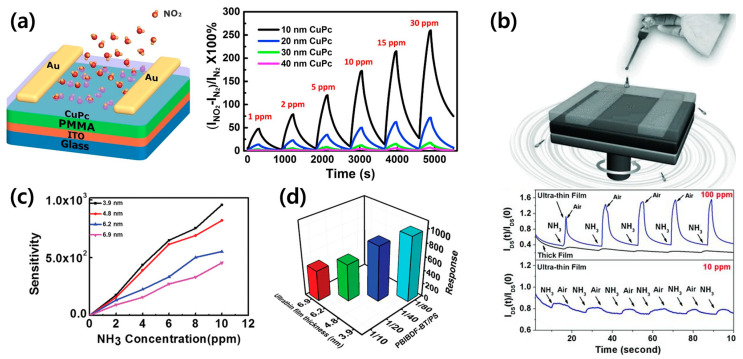
(**a**) Schematic illustration of copper phthalocyanine (CuPc) gas sensor detecting NO_2_ gas (**left**) and response curves of the four kinds of devices that have different film thicknesses to NO_2_ pulses (**right**). Reproduced with permission from Jiang et al. [38], Copyright © 2017, Elsevier. (**b**) Illustration of the on-the-fly dispensing spin-coating method (**top**) and I*_DSAT_* vs. time of thin-film and ultrathin-film transistors while detecting NH_3_ (**bottom**). Reproduced with permission from Zhang et al. [40], Copyright © 2013, WILEY-VCH. (**c**) Relationship between the sensitivity and NH_3_ concentration for different PBIBDF-BT films. (**d**) Relationship between PBIBDF-BT content, ultrathin film thickness, and sensitivity. Reproduced with permission from Chen et al. [41], Copyright © 2021, WILEY-VCH.

Typically, the performance of OFET-based gas sensors relies on the capability of the gas analyte to diffuse into the channel. Consequently, reducing the thickness of the channel where gas adsorption and diffusion occur can serve as an effective strategy to enhance the performance of gas sensors. By minimizing the diffusion route for gas molecules within the organic semiconductor, the ultrathin layer of the semiconductor enhances the device’s ability to sense. This shortened path can facilitate quicker interaction between the analyte gas molecules and the charge carriers in the charge–transport layer [17,38,39]. To investigate the effect of film thickness on gas-sensing capabilities, Jiang et al. fabricated CuPc-based OFETs with varying thicknesses ranging from 10 nm to 40 nm and compared the NO_2_ gas sensitivity at these different thicknesses [38]. As illustrated in Figure 3a on the right, it was observed that as the thickness of the CuPc film decreased from 40 nm to 10 nm, the sensitivity to gas increased from 7% to 241%.

Various methods, including deposition [42], spin-coating [43], bar-coating [25], and Langmuir–Schaefer [31], have been proposed to reduce the film thickness in OFETs. Specifically, the production of ultrathin films via solution-based methods continues to be a significant challenge, driving continuous research in the field [41]. Zhang et al. introduced an unusual spin-coating technique named “on-the-fly dispensing spin-coating” to create sub-10 nm ultrathin n-type OFETs [40]. By casting the solution while the substrate was rapidly rotating, they produced films, notably a 4nm ultrathin layer, which exhibited a gas response an order of magnitude greater than that of a 70 nm thick film, as shown in Figure 3b. Chen et al. utilized the semiconducting polymer bithiophene and PBIBDF-BT to fabricate an ultrathin film for gas sensors with a minimum thickness of 4 nm [41]. They employed vertical phase separation, an effective method for creating ultrathin films [44], and controlled the film thickness through solution concentration adjustments. To achieve self-assembled conjugated polymer films, they blended the material with polystyrene (PS), and the incompatibility between PBIBDF-BT and PS resulted in vertical phase separation after spin-coating. Subsequently, etching PS with ethyl acetate yielded the ultrathin PBIBDF-BT film. The thickness of the resulting PBIBDF-BT film varies according to the blend ratio of PBIBDF-BT to PS, with an increase in the PS ratio leading to thinner films. It was observed that a decrease in film thickness correlates with an increase in sensitivity to gas analytes, as shown in Figure 3c,d.

**Figure 4:**
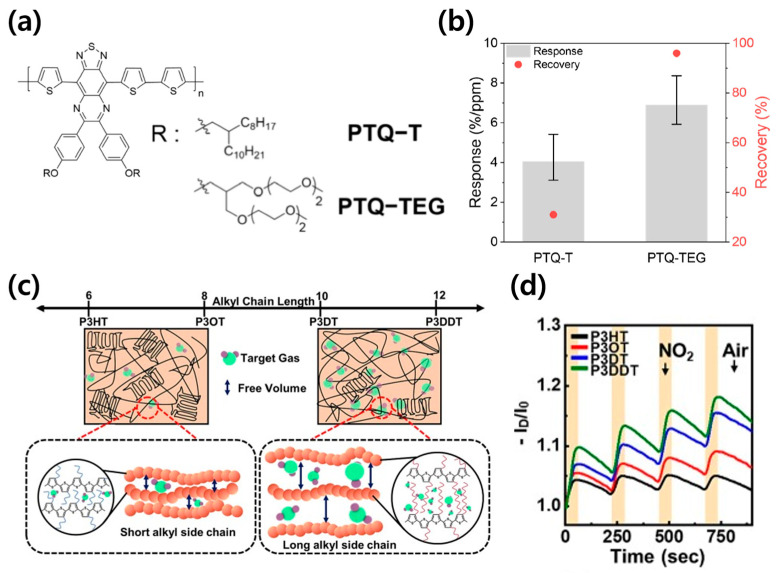
(**a**) Chemical structures of PTQ-T and PTQ-TEG. (**b**) NO_2_ detection and recovery characteristics of PTQ-T and PTQ-TEG. Reproduced with permission from Ahn et al. [46], Copyright © 2023, MDPI. (**c**) Schematic illustration delineates the distinct semicrystalline and amorphous regions within the film. (**d**) Repetitive gas-sensing curves of gas sensor devices upon exposure to 10 ppm NO_2_ from Hong et al. [47], Copyright © 2023, Elsevier.

In addition to enhancing the gas-sensing capabilities, considerable research has focused on modulating these characteristics via the manipulation of the material’s chemical structure [36]. To control thickness, the manipulation of the material’s chemical structure can be a vital approach to control free volume for enhanced sensitivity. Yang et al. reported the preparation of a porous pDPPBu-BT organic semiconductor (OSC) film, a polymer semiconductor, with thermal annealing at 240 °C. This thermal annealing process was used to expel gaseous isobutylene and convert tert-butoxycarboxyl groups into COOH, thereby augmenting NH_3_ gas sensitivity alongside the formation of a porous structure resulting from the isobutylene removal [45]. Ahn et al. explored the impact of side-chain variations on gas sensing by comparing PTQ-T, which features an alky chain, against PTQ-TEG, distinguished by its ethylene glycol-based side chain [46]. The ethylene glycol derivative side chain of PTQ-TEG exhibits greater flexibility relative to the more rigid alky chain of PTQ-T, thereby enhancing the free volume and facilitating the adsorption and desorption of gases (Figure 4a). This characteristic notably improves response and recovery times, as illustrated in Figure 4b. Furthermore, the inclusion of an oxygen atom in the ethylene glycol-based side chain enhances gas adsorption, especially for electron-withdrawing analytes such as NO_2_.

Hong et al. adjusted the side chain length of Poly(3-alkylthiophene) (P3AT) to facilitate the penetration of gas analytes into the OSC film [47]. The alkyl chain of P3AT influences various material properties, including mechanical characteristics, morphology, and intermolecular interactions. While longer alkyl chains are generally known to decrease charge transport efficiency [13], the free volume generated by long side chains can enhance analyte penetration, potentially improving gas sensor performance (Figure 4c). Long alkyl chains can form an insulating barrier between the conjugated backbone and the electrode, hindering the injection of charge carriers and potentially degrading the electrical properties of the OFET. However, Poly(3-dodecylthiophene) (P3DDT), which possesses the longest alkyl side chain, demonstrated superior NO_2_-sensing properties, with a sensitivity of 0.45%/ppm and a limit of detection (LOD) value of 0.26 ppt, approximately twice as effective as P3HT (Figure 4d). Thus, adjusting the length of the side chain can facilitate gas diffusion, enhancing sensor performance. Additionally, the fabricated P3DDT FET exhibited superior mechanical flexibility compared to the P3AT FETs with shorter side-chain lengths.

## 4. Porous Structure for Enhanced Sensitivity

**Figure 5:**
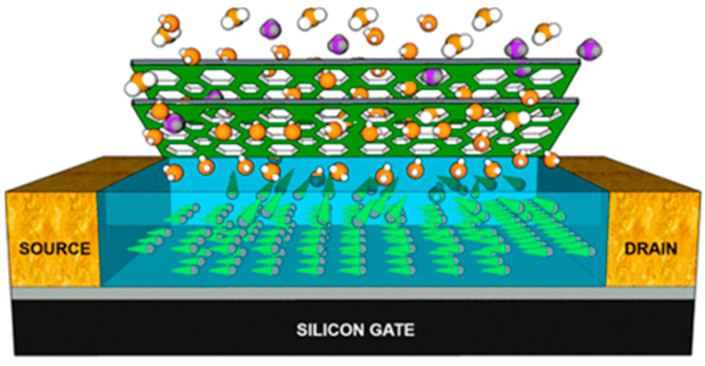
Schematic illustration of OFET gas sensor based on porous organic semiconductor. Reproduced with permission from Yuvaraja et al. [53], Copyright © 2020 American Chemical Society.

Porous structures are advantageous for maintaining the transport pathway of charge carriers while providing passages for the diffusion of target gas molecules (see Figure 5). Consequently, field-effect charge carriers near the gate dielectric are affected by the diffused gas molecules, leading to an increase in response and recovery during gas detection. The porous structure (so called, breath figure) needs to be finely constructed to maintain charge carrier mobility [48]. There are several strategies to construct porous structures. One method involves using spontaneous self-assembly during the solution process [49]. Thin film de-wetting could be utilized to directly fabricate the porous film [50]. Another approach involves using added solvents and polymers to facilitate the formation of porous structures [51]. Alternatively, unwanted regions can be removed using physical or chemical etching methods. The etching method is preferable for selecting material types. Both thermally evaporated organic semiconductors and solution-processed ones can be used for the formation of porous structures via etching [52]. In the next section, we will introduce representative works on the formation of porous structures for sensitive OFET-based gas sensors.

### 4.1. Self-Assembled Porous Structure

Research in the field has been directed towards manipulating the morphology or microstructure of OSC films by employing additive or blend solutions to augment the crystallinity and uniformity of the film coverage. The blend approach is quite beneficial for the formation of multicomponent films via spontaneous self-assembly and phase separation [54]. By using organic semiconductor/insulating polymer blends, OFET performance could be enhanced in several aspects. Predominantly, insulating polymers such as polystyrene have been identified as enhancing both the stability and mechanical properties of OFETs when integrated with diverse semiconductors. The control of solubility and surface energy plays a critical role in vertical phase separation and the formation of ultrathin semiconducting films on polystyrene. However, the performance of solution-processed OFETs is influenced by processing conditions such as spin-coating time. Investigations by Na et al. into polythiophene films have shown that the amount of residual solvent, contingent upon the spin-coating time, affects the electrical properties [55]. Optimal residual solvent levels were found to enhance the ordering of polythiophene molecules, crucial for modulating the crystallization rate and molecular orientation. Such adjustments are instrumental in determining the OSC film morphology. The morphological characteristics of the OSC film play a pivotal role in dictating the performance of gas sensors, as they directly influence gas diffusion rates and the extent of the contact area between the sensor and gas molecules [36].

**Figure 6:**
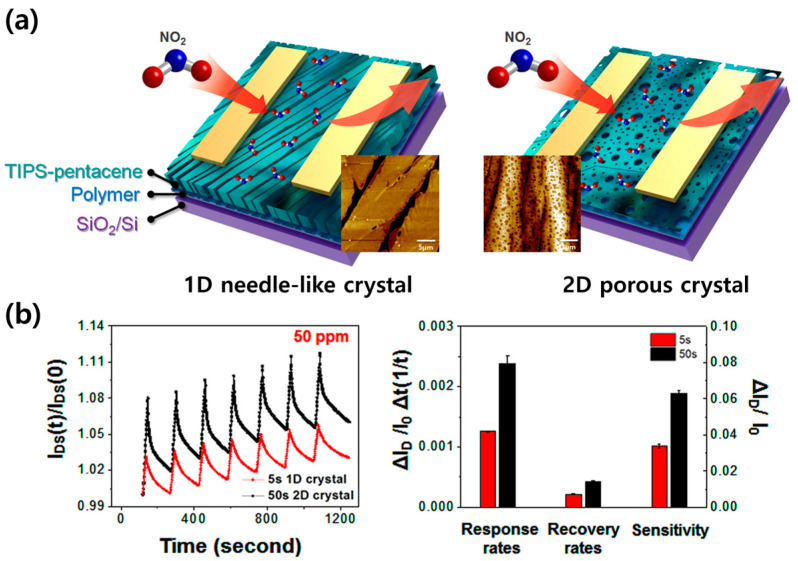
(**a**) Schematic illustration of TIPS-pentacene/PS OFETs in different spinning times. (**b**) **Left**: Repetitive sensing curves of OFET gas sensors based on the blend films upon exposure to successive pulses of NO_2_ (50 ppm) and N_2_. **Right**: Sensing parameters of TIPS-pentacene/PS sensors upon exposure to NO_2_ (50 ppm) and N_2_. All sensing experiments were carried out at V*_GS_* = −10 V and V*_DS_* = −10 V, respectively. Reproduced with permission from Lee et al. [56], Copyright © 2019, Springer Nature.

Lee et al. developed a method to produce porous OSC films by blending TIPS pentacene, a crystalline low-molecular-weight semiconductor, with polystyrene, an insulating polymer [56]. The morphology of TIPS pentacene can exhibit diverse structural configurations contingent upon the matrix in which it is embedded [57]. TIPS pentacene blended with polystyrene, when subjected to a brief spin-coating time, exhibits a predilection for a one-dimensional (1D) growth mode due to the abundance of residual solvent. This process initiates crystallization from the edges, culminating in the formation of 1D needle-like crystals. Conversely, an extended spin-coating time results in a reduction in residual solvent quantity, thereby facilitating the random nucleation of spherulites that leads to the development of two-dimensional (2D) porous crystals [58]. The characterization of surface microstructures, as illustrated in Figure 6a, demonstrates that 1D crystals are characterized by large-scale inter-crystal gaps, while 2D crystals feature a high density of voids. These morphological differences significantly impact the gas-sensing properties. As shown in Figure 6b, OFETs with a 2D crystal structure, produced through prolonged spin-coating processes, exhibited over twice the response, recovery, and sensitivity compared to those with a 1D crystal structure, which were fabricated with a shorter spin-coating time. This enhanced performance is attributed to the porous structure of 2D crystals, which facilitates the facile passage of gas molecules through the channel region. It is thereby confirmed that within organic semiconductor/insulating polymer blend systems, the optimization of spin-coating time allows for the control of OSC film morphology and microstructure, thereby enabling the regulation of device performance through the strategic manipulation of processing conditions.

In the fabrication of an organic semiconductor film by manipulating the coating dynamics of a blend solution, the formation of a porous structure is often challenged by the occurrence of aggregation, which is attributed to the pronounced molecular interactions during the solvent-annealing phase. This aggregation complicates the pore creation process. Dong et al. employed water to fabricate honeycomb-structured microporous films [59]. Research on nature-inspired breath figure models, which do not utilize lithography or etching processes, has been reported [60]. Notably, the formation of well-defined pore structures in films utilizing polystyrene has been reported [61]. Zhang et al. developed a porous OSC film employing polystyrene through the integration of an organic semiconductor/insulating polymer blend system alongside the breath figure model [62]. Conducting the spin-coating process for films in a high-humidity environment results in water condensation on the film surface. Subsequent thermal annealing to evaporate this water leads to the formation of a microporous film. The porosity of this microstructure varies with the relative humidity (RH%). As the RH value decreases, less water condensation occurs, culminating in the development of a denser film. It has been confirmed that OFETs fabricated through the breath figure model, despite possessing a porous structure with micropores, exhibited negligible variation in field-effect mobility.

**Figure 7:**
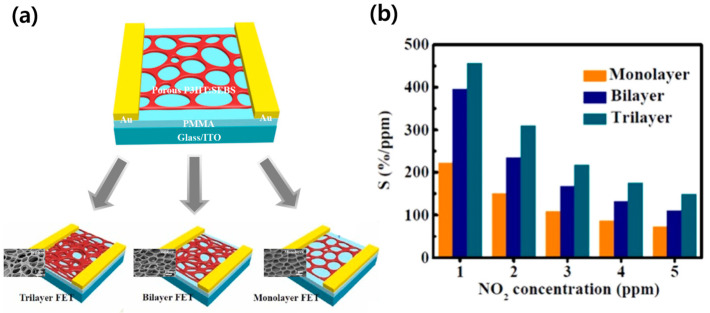
(**a**) Schematic illustration of OSC films by breath figure model. (**b**) Sensitivity of multilayer porous OFETs under various concentrations of NO_2_. Reproduced with permission from Gao et al. [63], Copyright © 2022, Elsevier.

The primary motivation for rendering porous gas sensors is to facilitate gas diffusion and increase the contact area. Transforming the conductive channel with which gas molecules interact into a three-dimensional structure is a strategic approach to enhancing gas sensor performance. Gao et al. fabricated a P3HT film and enhanced the gas response by stacking OSC films in multiple layers [63]. Each layer was produced by utilizing water condensation that occurs during spin-coating in a high-humidity environment, and the OFETs were constructed as monolayer, bilayer, or trilayer based on the number of layers stacked. Scanning Electron Microscopy (SEM) verified the formation of a three-dimensional porous structure (Figure 7a). OFETs with a porous multilayer configuration have a larger gas adsorption surface area compared to monolayers, enabling more sensitive detection of current changes due to analytes. In fact, gas-sensing properties varied with the stacked layers; for NO_2_ gas, at concentrations of 1~5 ppm, trilayer OFETs exhibited over twice the sensitivity compared to monolayer OFETs (Figure 7b). In addition, OFETs with porous multilayer films exhibited excellent mechanical performance compared to the dense film.

Beyond utilizing water condensation to create porous structures, water can also serve as a nonsolvent to fabricate porous films. Guillen et al. employed a combination of polymer, solvent, and nonsolvent to generate porous films, discovering that the morphology varies based on the miscibility between the nonsolvent and solvent. When the miscibility between solvent and nonsolvent is high, a rapid solvent–nonsolvent exchange occurs, resulting in the formation of a finger-like morphology. Conversely, if the miscibility between solvent and nonsolvent is poor, the solvent–nonsolvent exchange happens more slowly, leading to the development of a sponge-like morphology [64].

**Figure 8:**
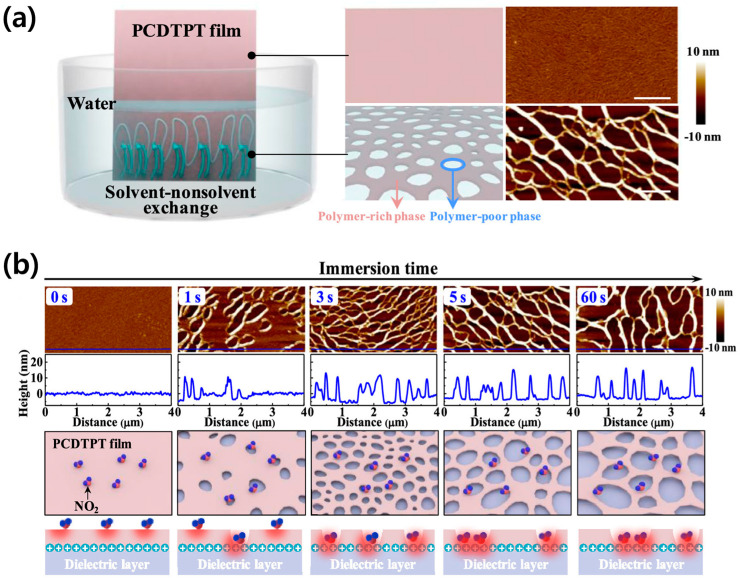
(**a**) Immersing a spin-coated, dense PCDTPT film into deionized water for a few seconds and then taking it out. Comparison of the schematic illustrations and AFM images of the initial and immersed PCDTPT film. (**b**) Influence of immersion time on the morphology of ultrathin porous polymer films. AFM images (4 μm × 2 μm) and height curves of ultrathin porous PCDTPT films with different immersion times. Reproduced with permission from Liang et al. [65], Copyright © 2020, American Chemical Society.

Liang et al. fabricated a porous PCDTPT film utilizing a solvent–nonsolvent exchange process [65]. Chlorobenzene was employed as the solvent to dissolve the polymer, and deionized water (DI water) was used as the nonsolvent. Upon spin-coating, the polymer solution onto a substrate and immediately immersing it in water, the initially dense film transforms into a porous structure (Figure 8a). The resultant PCDTPT polymer film acquires a cobweb-like appearance with thin, irregular pores. The size of these pores varies depending on the duration the film is submerged in the DI water nonsolvent; longer exposure times lead to larger pores (Figure 8b). The control of solubility parameters can change the dimensions of the porous structure. Thus, a solvent–nonsolvent exchange offers a direct means of controlling morphology. As the size of the pores increases, gas diffusion becomes more favorable; however, this can lead to a deterioration in the performance of OFET devices, necessitating the careful consideration of this effect.

### 4.2. Porous Semiconducting Polymer via Etching

**Figure 9:**
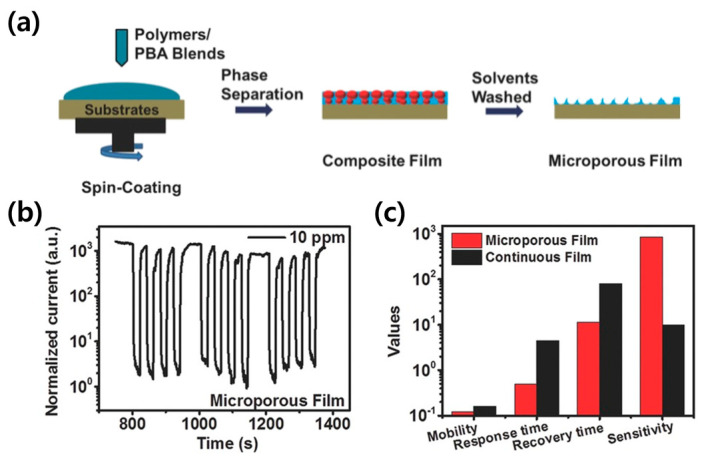
(**a**) Fabrication process: the dropping of the PBIBDF-BT/PBA blend on the substrate, then the phase separation of the polymer/PBA blends, and the obtained PBIBDF-BT film after washing with solvent. (**b**) Cyclic test performance of the sensors based on the PBIBDF-BT microporous film. (**c**) Histogram showing the properties of the microporous- and continuous-film-based sensors. Reproduced with permission from Wang et al. [66], Copyright © 2016, WILEY-VCH.

One of the most straightforward methods for creating a porous structure is controlling film surface morphology through physical etching. The etching process entails removing a portion of the surface layer of the fabricated OSC film, thereby enlarging the area available for gas analyte adsorption. Furthermore, it reduces the layer’s thickness, bringing it closer to the conductive channel, which leads to an increase in response and enhanced recovery capabilities. Wang et al. fabricated a composite film by blending a conjugated polymer with poly(1,4-butylene adipate) (PBA) and applying spin- coating, followed by etching away the PBA to create a microporous film (Figure 9a) [66]. The microporous film, from which PBA was etched away, exhibited more than 800 times of current change upon exposure to NH_3_ (Figure 9b). It was compared to continuous films; there was an enhancement in sensitivity exceeding 200 times (Figure 9c).

**Figure 10:**
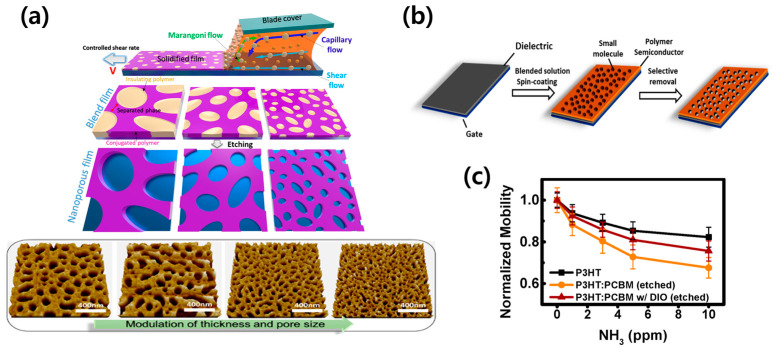
(**a**) Schematic illustration of the modulation of the surface morphology of ultrathin nanoporous P3HT films using the SAPS method (**top**). Atomic force microscopy (AFM) images of shear-coated nanoporous P3HT films for various shear rates (**bottom**). Reproduced with permission from Tran et al. [69], Copyright © 2022, American Chemical Society. (**b**) Illustrative diagram presenting the fabrication of morphology-controlled polymer films by selective dissolution of a small molecular component. (**c**) Normalized hole mobility of devices according to various NH_3_ concentrations. Reproduced with permission from Park et al. [67], Copyright © 2019, MDPI.

The etching method, leveraging differences in solubility, can be applied to materials beyond polymers. Park et al. utilized small molecules, instead of polymers, as the etching substance to create pores that allow for gas ingress and egress [67]. After spin-coating a blend of P3HT and phenyl-C61-butyric acid methyl ester (PCBM), n-butyl acetate (BA) was employed to selectively etch PCBM (Figure 10b). This enhanced the response of the P3HT:PCBM film compared to standalone P3HT film, as shown in Figure 10c. The solvent-based etching technique, applied post-blend solution coating, faces difficulties in pore size regulation, highlighting the need for additional investigation [68]. Tran et al. fabricated an OFET gas sensor with a nanoporous film through shear coating and utilized shearing-assisted phase separation (SAPS) to adjust pore size by varying coating speeds [69]. When coating a blend solution of P3HT and PS using the SAPS method, the shear rate can be varied from 0.5 mm/s to 40 mm/s to achieve pore sizes ranging from 90 nm to 500 nm. Below a shear rate of 4 mm/s, an increase in rate leads to a decrease in pore size; however, beyond 4 mm/s, an increase in shear rate results in larger pores. At shear rates above 40 mm/s, the pore size exceeds 500 nm, larger than those obtained via spin-coating (Figure 10a). They observed that films sheared at 4 mm/s exhibited over 70% response to gas, and with increasing shear rates, the sensitivity to gas analytes decreased. This demonstrates that by simply adjusting the shear rate, it is possible to control the pore size of the porous structure, thereby enhancing gas-sensing properties.

### 4.3. Porous Evaporated Semiconductor via Etching

The fabrication of OSC films from small-molecule semiconductors like pentacene or Dinaphtho [2,3-b:2′,3′-f] thieno [3,2-b] thiophene (DNTT) through thermal evaporation is a well-established method. Small-molecule semiconductors form crystals through π-π interactions as molecules pack together, allowing for the control of the desired OSC film morphology by managing these interactions. Typically, the insertion of a self-assembly monolayer (SAM) between the semiconductor layer and the substrate can regulate the nanoscale ordering and interface characteristics of the organic semiconductor layer [70]. Lee et al. demonstrated that the properties of the interface could be controlled by adjusting the deposition temperature [71].

Control over deposition temperature for interface management serves as an effective strategy for morphology control by inducing the selective adsorption of molecules. Kang et al. leveraged temperature-controlled deposition to induce the 2D crystal growth of pentacene using a rubbery template with a smooth surface [72]. A template of m-bis(triphenylsilyl)benzene (TSB3), a small molecule dielectric compound with a low glass transition temperature (T_g_) of approximately 33 °C, was deposited on an OTS-treated substrate, followed by the deposition of the semiconductor pentacene to create a porous structure OSC film. Due to the low surface energy of the smooth OTS layer, when TSB3 is deposited, the substrate is in a state above TSB3’s T_g_, preventing full coverage by the rubbery state TSB3 and leading to some agglomeration during film deposition. Depositing pentacene on the dewetted TSB3 film results in preferential adsorption onto TSB3 due to its higher interaction energy requirement with OTS. Consequently, pentacene deposits follow the morphology of the underlying TSB3, facilitating the creation of a porous structure OFET that allows for easy analyte penetration. On the other hand, the deposition rate significantly influences the kinetics of nucleation and growth processes during film formation. Higher deposition rates can result in higher nucleation density and hinder the growth of large crystalline domains, thus favoring the formation of porous structures with smaller pore sizes and higher surface areas [73].

**Figure 11:**
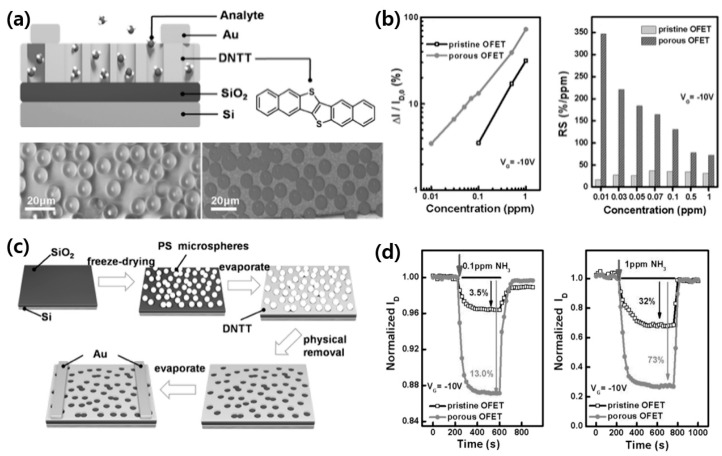
(**a**) The device structure of the porous OFET-based sensors and the molecular structure of DNTT (top). Optical images of a porous DNTT film (**bottom left**) with polystyrene microspheres and (**bottom right**) after removing polystyrene microspheres. (**b**) (**left**) The I*_D_* changes in the pristine and porous OFETs with different concentrations of NH_3_. (**right**) The relative sensitivity (RS) of the pristine and porous OFET-based sensors in response to various concentrations of NH_3_ vapor. (**c**) The fabrication procedure of the porous OFET-based sensors. (**d**) Sensing responses of the pristine and porous OFETs, with air acting as background vapor. Compared I*_D_* changes in the two OFETs in response to (**left**) 0.1 ppm NH_3_ and (**right**) 1 ppm NH_3_. Reproduced with permission from Lu et al. [74], Copyright © 2017, WILEY-VCH.

While it is possible to pre-fabricate a porous template for semiconductor layer stacking, an alternative approach involves pre-patterning pores on the template before depositing the semiconductor layer. Lu et al. fabricated a porous structure OFET gas sensor using DNTT and PS, as shown in Figure 11a [74]. Using the vacuum freeze-drying method, polystyrene microspheres were deposited onto the substrate, followed by the thermal evaporation of DNTT. Subsequently, the polystyrene microspheres were physically removed using adhesive tape, creating a porous DNTT OSC film (Figure 11c). This process resulted in the formation of pores approximately 10 μm in size where the PS had been removed. A comparison between porous OSC films and dense pristine OSC films (Figure 11b,d) revealed that the porous OFETs were capable of sensing gas concentrations starting from 10 ppb, whereas the pristine OFETs could only detect gas concentrations above 0.1 ppm.

Table 1 summarizes performance of OFET-based gas sensors in this review.

## 5. Conclusions and Future Perspective

In this review, we have explored the operating mechanisms and strategies for enhancing sensitivity in OFET-based gas sensors. By focusing on factors like carrier mobility, charge carrier density, and gas diffusion, we have highlighted the importance of controlling thickness and free volume within the organic semiconductor layer. These strategies, along with manipulating material chemical structures, have shown promise in significantly improving sensor performance. Porous structures have emerged as a key avenue for enhancing sensitivity in OFET-based gas sensors. These structures facilitate the diffusion of target gas molecules while maintaining the transport pathway for charge carriers. Through innovative fabrication techniques such as self-assembly methods and etching, researchers have been able to create porous structures with tailored morphologies, leading to increased response and recovery during gas detection. The optimization of processing conditions, including spin-coating time and solvent selection, has played a crucial role in controlling film morphology and microstructure, thereby enabling the precise regulation of device performance. Additionally, physical etching techniques have been explored to create porous structures, effectively enlarging the surface area available for gas analyte adsorption.

There are several advantages to methods for constructing porous structures in OFETs. For instance, in solution processes, the ability to regulate solvent evaporation rates during film deposition offers precise control over pore formation, facilitating the fine-tuning of pore sizes without requiring additional template materials. Furthermore, the availability of solution-based techniques enables easy deposition onto flexible substrates, and their compatibility with roll-to-roll processing methods like slot–die coating or gravure printing ensures high throughput and scalability for the mass production of porous OFETs [75]. These methods can coat large areas rapidly and continuously, making them suitable for industrial-scale manufacturing. Despite their advantages, certain limitations exist. Some techniques may struggle to achieve extremely small or large pore sizes, hindering optimization for specific device requirements. Additionally, methods requiring precise control over parameters like solvent evaporation rates or assembly conditions can be complex and require meticulous optimization, increasing the difficulty of fabrication [76]. Moreover, issues such as uneven surfaces, roughness, or the formation of defects within the porous structure can adversely affect OFET device performance. Thus, the development of OFET-based gas sensors relies on optimizing sensor properties by addressing these issues.

The sensing properties of OFET gas sensors could be influenced by the structure’s geometry. Recent work has revealed that the placement of the source–drain electrodes in OFET gas sensors determines whether the structure is top contact or bottom contact [43]. The top-contact structure features a longer injection path, which is more susceptible to the influence of charge carriers from adsorbed NO_2_ molecules, thereby enhancing its sensing performance. In contrast, the bottom-contact structure, with a shorter injection pathway [77], exhibits lower sensitivity. Consequently, the response in the top-contact structure tends to be higher compared to that observed in the bottom-contact structure. Further works on optimizing device geometry can increase the sensor performance. Because the organic semiconductor has several drawbacks, the performance of OFET gas sensors could be enhanced by adopting 2-dimensional materials such as graphene [78,79]. This hybrid-type sensor can widen the sensing capability of various types of sensors. In particular, OFET performance is greatly affected by the humidity in atmospheric conditions. The degradation of sensor performance could be reduced by the choice of inorganic materials, which can serve as erasers of water molecules. These advancements pave the way for the development of highly sensitive and selective OFET-based gas sensors with potential applications in environmental monitoring, industrial safety, and healthcare [80,81].

## Figures and Tables

**Table 1:** A summary of OFET-based gas sensors in this review.

Method	Processing	Sensing Material	Analyte	Detection Range	Detection Limit	Sensitivity [%/ppm]	Refs.
Thickness control	Spin coating	CuPc	NO_2_	1~30 ppm	-		[40]
Thickness control	Spin coating	PBIBDF-BT	NH_3_	0~10 ppm	2 ppm	-	[41]
Side Chain control	Spin coating	PTQ-TEG	NO_2_	50 ppm	1.59 ppb	6.9	[46]
Side Chain control	Spin coating	P3DDT	NO_2_	10~50 ppm	0.26 ppt	0.45	[47]
Self-assembled porous structure	Spin coating	TIPS-pentacene/PS	NO_2_	1~50 ppm	-	~2	[56]
Breath figure method	Spin coating	P3HT/PS	NO_2_	0~20 ppm	-	48.2	[62]
Breath figure method	Spin coating	C8-BTBT/PS	NH_3_	0~20 ppm	-	12.5	[62]
Breath figure method	Spin coating	N2200/PS	NH_3_	0~20 ppm	-	~4.5	[62]
Multiple layered Breath figure model	Spin coating	P3HT	NO_2_	0.5~30 ppm	2.3 ppb	457	[63]
solvent–nonsolvent exchange	Spin coating	PCDTPT	NO_2_	0~30 ppm	<1 ppm	9.89 × 10^3^	[65]
Selective Etching	Spin coating	PBIBDF-BT	NH_3_	10 ppm	0.5 ppm		[66]
Selective Etching	Shear coating	P3HT/PS	NH_3_	0.5~30 ppm	0.5 ppm	7.02	[69]
Selective Etching	Spin coating	P3HT	NH_3_	10 ppm	1 ppm		[67]
Porous template	evaporation	DNTT	NH_3_	0~10 ppm	10 ppb	340	[74]

## Data Availability

The data presented in this study are available on request from the corresponding author.
